# Bidirectional Relationship between Depression and Cognitive Function: Evidence from a Psychosocial Intervention

**DOI:** 10.1093/geroni/igaf122.2032

**Published:** 2025-12-31

**Authors:** Lucy Xi Lu, Dara Kiu Yi Leung, Stephanie Ming Yin Wong, Wai Chi Chan, Gloria Hoi Yan Wong, Terry Lum

**Affiliations:** The University of Hong Kong, Kowloon, Hong Kong, China (People’s Republic); The University of Hong Kong, Kowloon, Hong Kong, China (People’s Republic); The University of Hong Kong, Kowloon, Hong Kong, China (People’s Republic); The University of Hong Kong, Kowloon, Hong Kong, China (People’s Republic); University of Reading, Reading, United Kingdom; The University of Hong Kong, Kowloon, Hong Kong, China (People’s Republic)

## Abstract

Depression is a modifiable predictor of dementia, but its underlying relationships with cognition is unclear. Most research studied their relationships in spontaneous contexts, lacking insight into their interactions during interventions. This research examined the longitudinal relationships between depressive symptoms (DS) and cognitive function (CF) across psychosocial intervention in older adults with intact cognition and mild cognitive impairment (MCI). Data were from JC JoyAge, a community stepped-care program for late-life mental health. Participants (≥ 60 years) with mild to moderate DS (n = 4448) completed assessments of DS (PHQ-9) and CF (MoCA 5-min) before and after the program. Bivariate latent change score models were computed to examine the interactions between DS and CF by modeling changes as latent variables and testing individual-level relationships, with multigroup modeling for comparisons. Participants experienced improvements in DS and CF. For both participants with intact cognition and MCI, higher baseline CF predicted greater reduction in DS (est = -0.04, se = 0.01, z = -3.83, p < .001), lower baseline DS predicted greater improvement in CF (est = -0.04, se = 0.01, z = -3.76, p < .001). Greater reduction in DS was associated with greater improvement in CF (est = -0.51, se = 0.11, z = -4.65, p < .001). The findings evidenced the bidirectional relationships between depressive symptoms and cognitive function, and showed their hand-in-hand improvements during psychosocial intervention. Results suggested the benefits of treating depressive symptoms for both mental health and cognitive function, no matter at the stages of intact cognition or MCI.

